# Estimating Fibrosity Scores of Plant-Based Meat Products from Images: A Deep Neural Network Approach

**DOI:** 10.3390/foods15040665

**Published:** 2026-02-12

**Authors:** Abdullah Aljishi, Shirin Sheikhizadeh, Sanjoy Das, Sajid Alavi

**Affiliations:** 1 Department of Electrical & Computer Engineering, Kansas State University, Manhattan, KS 66506, USA; 2 Department of Grain Science & Industry, Kansas State University, Manhattan, KS 66506, USA; shirinsheikhi@ksu.edu (S.S.); salavi@ksu.edu (S.A.)

**Keywords:** convolutional neural network, deep neural network, explainability, extrusion, fibrosity, machine learning, image processing, plant-based meat, regression, residual network

## Abstract

This paper proposes a deep neural network to estimate the fibrosities of plant-based meat product images. Images of varying fibrous microstructures were collected for this purpose, which were subject to spatial preprocessing and data enhancement. Their corresponding fibrosity scores were provided by two human experts. This data was used to train the network and to analyze its performance. Various statistical performance metrics were applied to evaluate the accuracy of the trained network’s estimated scores. It was found that the network performed significantly better when trained separately with fibrosity scores of each individual subject than with their combined scores, indicating that it was able to capture nuanced aspects of a subject’s perception. Another study was directed at explainability of the network’s estimates. Using standard software, a set of synthetic images of varying shapes and sizes were created as inputs to the network. Visual inspection of the output scores indicated that its estimates were influenced only by those features (i.e., food matrices and air cells) that were directly relevant to fibrosity, and not by extraneous factors.

## 1. Introduction

Extrusion is a common method for producing texturized vegetable proteins (TVP) or plant-based meat. Through the combined effects of heat, pressure, shear, and moisture, such plant-derived proteins are transformed through this process into fibrous, meat-like structures. This technology is valued not only for its ability to replicate the texture of meat, but also for its potential to deliver environmentally sustainable, high-protein foods. Diverse protein sources, such as soy, pea and hemp, have been used for TVP products, which underscores the adaptability of the extrusion process [1,2,3].

Microstructural properties are key factors that influence various textural signatures of the product, such as mechanical strength, chewiness, and springiness [4]. The degree of fibrosity (i.e., fibrousness) of TVP products is often evaluated subjectively from product images [2,5,6,7,8].

The underlying goal of this research is to develop a model that can objectively assess the fibrosities of plant-based products from input images. Such an automated scheme to provide numerical output scores would help in reducing—if not eliminating, the need for subjective human inspection during the extrusion process. Hence, this research is a major step towards the development of better structures and textures, as well as for process control automation, obviating the need for intermittent human inspection.

Research articles on methods that treat microstructural features of TVP in a similar objective manner have begun to appear. A review article on analytical approaches for assessing plant-based meat analogs, including microstructure analysis using image processing algorithms to obtain fiber index values, has been published in [9]. An automated image analysis method (i.e., Fiberlyzer) to quantify fibrosities of plant-based meats was proposed in [10]. Strong correlations between computed fiber scores and expert panel evaluations demonstrate the effectiveness of this approach, thereby illustrating how computer vision can be leveraged for objective assessment. A non-destructive, laser transmission method using computer vision to quantify the degrees of orientation in fibrous foods has been proposed in [11]. This technique was shown to reliably capture structural alignment—a feature associated with mechanical texture and consumer acceptance, thereby offering a more objective alternative to traditional visual inspection. The relationship between structural and mechanical anisotropy in plant-based meat products has also been examined [12]. This study, which draws on X-ray scattering and scanning electron microscopy, demonstrates how high protein content and controlled processing conditions promote fibrous alignment and mechanical strength. Microstructural anisotropy indices served as robust indicators of product quality in this research. More recently, an integrated framework to obtain TVP fibrosity scores from extrusion parameters, was explored in [13]. This approach, which involves machine learning and computer vision, can be adopted in realizing optimal process control in real time.

A deep neural network (DNN), such as that proposed in this research, is a trainable machine learning model that is roughly organized in the manner of the human cortex [14]. It consists of several layers of array processing. The first layer (input layer) acquires the DNN’s input, which is passed onto the next “hidden” layer. Each such hidden layer receives an input array from its immediately preceding layer and obtains an intermediate output that is supplied to the next layer. After several layers of processing, the final layer (output layer) produces the output of the DNN. The DNN’s weights, biases and other internal parameters can be iteratively optimized by means of a suitable learning algorithm. Classification and regression are the two broad categories of supervised learning. In classification, the DNN produces discretized outputs, while the outputs are continuous quantities in regression tasks. Since this research involves regression, the outputs of the proposed DNN are real quantities between 1 and 10 representing estimated fibrosity scores of input images. These images are obtained from plant-based meat products with varying textural attributes.

Recent years have witnessed an explosive growth in the popularity of DNNs. DNNs have been highly successful in a wide variety of applications, such as home automation [15], agriculture [16], large language models [17], cybersecurity [18], automated ground drones [19], automated traffic networks [20], defense [21], blockchains [22], and robotic control [23].

DNNs have been applied to various food-related image tasks, such as evaluating fish quality [24,25], predicting the soluble solid content of sweet potato [26], classifying tea leaf samples [27], classifying rice samples [28,29], and detecting cracks in wheat kernel images [29]. A significant amount of research attention is directed at gleaning coherent explanations from DNNs which are usually treated as black box models [30,31].

A residual network (ResNet) is a class of DNNs that was first proposed for image classification [32]. A unique feature of ResNets is the presence of residual connections. This feature allows a hidden layer to deliver its output simultaneously to two downstream layers. In general, ResNets incorporate several hidden layers with residual connections.

ResNets with 18, 34, 50, and 150 layers were investigated in [32], where they consistently outperformed traditional DNNs with up to 1000 layers. Theoretical treatment provides further insights for the superior performances of ResNets [33,34]. They have been successfully used in a wide variety of image processing [33,35]. ResNet architectures have also been adopted for food-related image processing. ResNets with 18 layers (ResNet-18) have been considered for such applications [29,36,37,38]. ResNets with 18 as well as 34 layers have been explored in [39]. Larger ResNets with 50 layers have been proposed elsewhere [40,41]. A ResNet with 101 layers has been studied [42]. In all these cases, ResNets were used for classification tasks. However, in a recently published article [43], the output of an open source ResNet-18 was explored for further statistical treatment also including regression analysis.

In this research, a ResNet-18 model that had been pre-trained for image classification [44] was suitably modified to perform regression. A new layer was incorporated into the DNN, while its input layer was enlarged to handle larger images. Using data collected for this investigation, a few layers of the original ResNet-18 were retrained for regression. This technique, called transfer learning, is used to curtail the needed training time [28,40]. Recent research reports the use of transfer learning for a similar application [45].

The next section describes at greater length the data collection methodology, image preprocessing, augmentation and human scoring of real images, the generation of synthetic images, as well as the layout and re-training of the 19 layered ResNet model that was developed as part of this research.

## 2. Methods

### 2.1. Generation of TVP Products

Three fava bean concentrate-based (45%) formulations, each containing soy protein concentrate (11%) but different sources of complementary plant proteins (44%), viz., pea protein isolate, soy protein isolate or wheat gluten, were extruded under different processing conditions to generate TVP products with varying fibrous microstructures. The protein contents of fava bean concentrate (Ingredion, Westchester, IL, USA) and soy protein concentrate (ADM, Quincy, IL, USA) were 60% and 72%. In pea protein isolate (Puris, Minneapolis, MN, USA), soy protein isolate (ADM, Quincy, IL, USA) and wheat gluten (Royal Ingredients Group, Alkmaar, The Netherlands), the contents were 80%, 90% and 82%. Thus, the net protein contents were in the range of 70.1–74.5% in the three formulations. The selection and combination of ingredients were informed by prior work [1], emphasizing the role of protein type and ratio in controlling the structural properties of the extruded plant-based meat products.

The three formulations were processed using a pilot-scale co-rotating twin-screw extruder (TX-52, Wenger Manufacturing, Sabetha, KS, USA), with a 52 mm screw diameter and a length to diameter (L/D) ratio of 19.5. The extruder comprised four-barrel zones, with temperatures set to 30 °C, 50 °C, 80 °C, and 110 °C from the feed section to the die end. A constant feed rate of 50 kg/h was maintained for all treatments, and the screw speed was fixed at 450 rpm. An aggressive screw configuration was selected, including cut flight, reverse, and kneading block elements, to achieve high shear and mechanical energy input necessary for protein texturization [1].

A venturi die of thickness ¼ inch was used to enhance shearing before the material flowed through dual ¼ inch outlet dies. The extrudate was then cut into pieces using a rotary knife system with three blades. The cut-extruded products were conveyed to a dual pass dryer (Series 4800, Wenger Manufacturing, Sabetha, KS, USA) and dried at 113 °C for 14 min, followed by 5 min of ambient air cooling. Each formulation was processed under different extrusion in-barrel moisture content conditions (ranging from 29.2 to 40.9% wet basis), resulting in six distinct plant-based meat extrusion treatments and corresponding products. Product collection from the dryer was done at various times during the processing, from which 63 TVP pieces were selected, which were spread over the six treatments.

These samples were utilized for image analysis, thereafter, to investigate the accuracy of the proposed DNN in estimating the samples’ fibrosity scores.

### 2.2. Data Acquisition: Real Images

To analyze the internal structure of the extruded textured vegetable protein (TVP) products, high-resolution macro images were captured using a Nikon D750 digital camera equipped with a 105 mm macro lens and SB-R200 wireless remote flash (Tokyo, Japan). The imaging setup included a Kaiser Copy Stand RS1, two Dracast Camlux Pro LED light panels, and an 18% grey card as the background to ensure standardized lighting and color balance. Image acquisition was conducted using CaptureOne software (version 10.1.1.5, Phase One, Copenhagen, Denmark).

Prior to imaging, dried TVP samples were rehydrated in tap water for 30 min and then drained for a duration of five minutes. Out of a total of 63 TVP samples that were collected, 18 hydrated pieces were sliced both longitudinally and transversely (relative to the direction of extrusion) in order to expose internal structural features. This procedure, which allowed for the visual inspection of cross-linking and layering densities in different directions, was used to capture 36 images for subsequent analysis. The remaining TVP samples were horizontally sliced, thus providing 45 additional time-series image samples. A total of K=81 raw images were acquired in this manner.

Each image $I_{i}^{raw}$, (i=1,…,K) was in the form of a three-dimensional array of 32-bit unsigned pixels, i.e., $I_{i}^{raw}\in {\left\{0\ldots 255\right\}}^{M\times N\times 3}$, where M×N is the raw image size. Additionally, several synthetic images were created using image software. The real images were subject to further treatment as outlined next.

### 2.3. Data Preparation: Real Images

**Figure 1:**
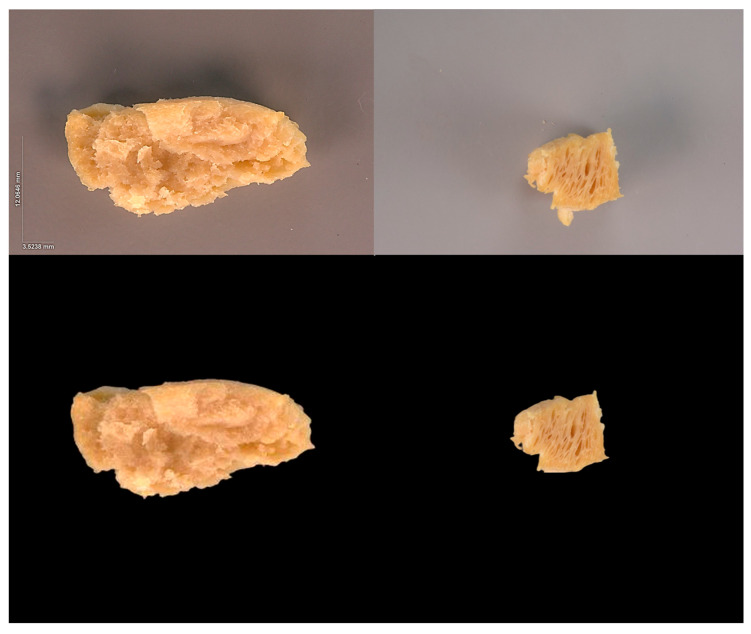
**Real Image Preprocessing.** Two raw (**top**) and preprocessed (**bottom**) image samples are shown.

In order to isolate the ‘figure’ (i.e., the portion showing the food matrix) from the ‘background’ in each of the of K=81 raw images, a suitable threshold was applied in a pixelwise manner, and those below it were recolored with black in order to remove background clutter and isolate the region’s relevant ‘figure’ portion. The raw images $I_{i}^{raw}$ (i=1,…,K) were zero-padded so that they were square shaped with identical horizontal and vertical sizes $I_{i}\in {\left\{0,\ldots ,255\right\}}^{6032\times 6032\times 3}$. The relevant ‘figure’ of each image was translated along the x and y axes so that its centroid coincided with the image’s mid-point. This preprocessing step ensured that all K images were properly aligned. The preprocessed images’ horizontal and vertical sizes of 6032 pixels, which was ~25% that of the largest raw image, were small enough to serve as DNN inputs while also retaining all textural features. For comparison, in another application also involving plant-based meat analogs [45], the input images to a ResNet-18 were of size 224×224×3, i.e., an order of magnitude smaller than the present ones. Figure 1 shows two examples of raw images (top row) along with the corresponding preprocessed images (bottom row).

**Figure 2:**
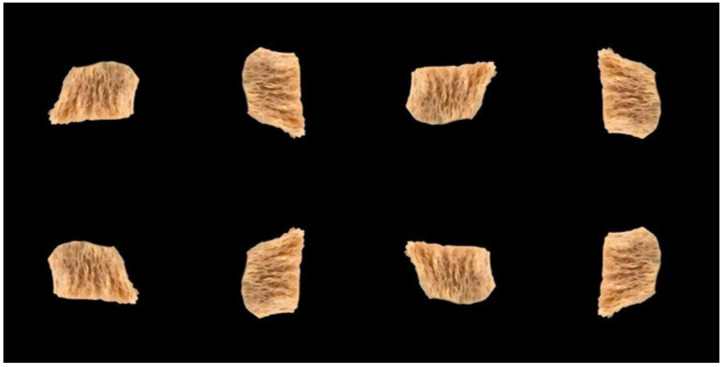
**Real Image Augmentation**. Shown are the eight (L=8) spatial orientations of a sample image. The orientations are the original (i.e., 0° rotation); rotations of 90°, 180°, and 270° (**top row**); the reflection; as well as its rotated counterparts.

Since the number of samples was relatively sparse, spatial data augmentation was carried out [46] before training the DNN. Similar spatial operations are routinely used for traditional image augmentation [47]. Image augmentation methods have also been applied in food processing [48]. In this research, each processed image $I_{i}$ was subject to reflection (i.e., mirror image), as well as rotations of 0°, 90°, 180°, and 270°. These spatial operations generated L=8 samples from each $I_{i}$, resulting in a total of K×L=648 input samples. Each such re-oriented image will be represented as $I_{i,l}$ (l=1,…,L). Figure 2 shows all eight spatial orientations of an image. It can be observed in the figure that the centroids in all the L=8 images are either in perfect alignment or have a discrepancy of ±1 pixels.

### 2.4. Human Scoring: Real Images

The images were assessed for quality by two human experts (A and B) with substantial academic research experience in plant-based meat production. Each subject provided a score for each image, on a scale of 1 through 10, with a higher score indicating more fibrosity. In order to account for discrepancies in human judgment, scores were obtained through multiple sessions that were scheduled on different dates. A total of six sessions were conducted (two with subject A, four with subject B).

A MATLAB program was developed for this purpose. During each session, the program displayed on screen for a subject all K=81 images. They were displayed sequentially but in random order. Furthermore, for each image $I_{i}$ only one was picked randomly and without repetition from the L=8 possible orientations $I_{i,l}$. The subject was provided online keyboard entry and a score $s_{i,p}^{X}\in [1,10]$, where X∈{A,B} is a subject and $p=1,\ldots ,P^{X}$ is a session index, so that $P^{A}=2$, and $P^{B}=4$. The mean score ${\bar{s}}_{i}^{X}$ of each image $I_{i}$ was obtained separately for each subject X as

(1) $$\begin{array}{c} {\bar{s}}_{i}^{X}=\frac{1}{P^{X}}\sum_{p=1}^{P^{X}}s_{i,p}^{X}.\; \end{array}$$

For each image $I_{i}$, the set of individual session scores ${\left\{s_{i,p}^{X}\right\}}_{p=1\ldots P^{X}}$, as well as their mean score ${\bar{s}}_{i}^{X}$, were stored as the first three fields of the datasets $S^{A}$ and $S^{B}$.

Due to the limited number of sessions per subject, not all image-orientation pairs could be manually scored during the interactive sessions. All such pairs were assigned scores randomly from the corresponding scored pairs. Moreover, preliminary simulations indicated that dissociating the L orientations of the images from their manual scores imparted robustness to the trained DNN. Specifically, for each image and each orientation $I_{i,l}$, a score $s_{i,p}^{X}$ was drawn randomly and without replacement from the existing ones, $s_{i,1}^{X}$ through $s_{i,P^{X}}^{X}$. The session index $p=1,\ldots ,P^{X}$ was a uniformly distributed random number. Accordingly, each $I_{i,l}$ was assigned an individual human score $s_{i,l}^{X}\in {\left\{s_{i,p}^{X}\right\}}_{p=1\ldots P^{X}}$. The sets of pairs ${\left\{I_{i,l},s_{i,l}^{X}\right\}}_{l=1\ldots L}$ were included as the fourth and final fields in the datasets $S^{A}$ and $S^{B}$.

In this manner two complete sets of data, $S^{A}$ and $S^{B}$, were obtained. As a reference for subsequent sections, the generic format is as shown below:

(2) $$\begin{array}{c} S^{X}={\left\{I_{i},{\left\{s_{i,p}^{X}\right\}}_{p=1\ldots P^{X}},\;{\bar{s}}_{i}^{X},\;{\left\{I_{i,l},s_{i,l}^{X}\right\}}_{l=1\ldots L}\right\}}_{i=1\ldots K}.\; \end{array}$$

The superscript X refers to a subject, so that X∈{A,B}. The subscript $p=1,\ldots ,P^{X}$ is a session index, while the subscript l=1,…,L denotes an orientation. The redundancy in Equation (2) is intended for clarity and does not reflect the true format of the data that was stored in computer memory.

### 2.5. Data Preparation: Synthetic Images

The immediate purpose of synthesizing additional images was to ensure that the trained DNN was free of inductive bias [49], i.e., that its output estimates were independent of any extraneous features in the real image samples. Inductive bias in DNNs, where they learn to pick artificial cues from their training datasets, has been long identified as a problem in supervised learning tasks [49,50,51]. Although bias in homogeneous DNNs has been extensively studied (Vardi, 2023), it is not well understood in the context of heterogeneous DNNs, including ResNets [52].

More broadly, synthetic images would allow the DNN’s output estimation to be more interpretable (explainable). Explainable AI is a topic of significant interest [31,36,53]. Explainable AI methods have been explored in image processing [54,55,56].

To ensure that the DNN was not sensitive to irrelevant image features, and to render its estimation more interpretable, a total of K=30 synthetic images were created. Each image was assigned a unique index number between 1 and 30. Based on their shapes, the synthetic images fell under the following four categories, (*i*) “large circle” (LC), (*ii*) “box” (BO), (*iii*) “ellipse” (EL), and (*iv*) “small circle” (SC).

**Figure 3:**
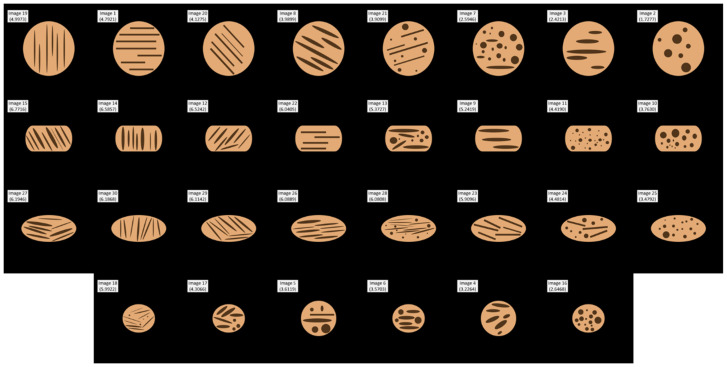
**Synthetic Images.** All 30 synthetic images are shown. The four image categories are “large circle” (**row 1**), “box” (**row 2**), “ellipse” (**row 3**), and “small circle” (**row 4**). The images in each row are arranged according their estimated scores, from best (**left**) to worst (**right**). The white rectangular box at the top left of each image shows the image number (1–30) and its estimated fibrosity score (averaged over all L=32 spatial orientations). DNN input images (addressed in Section 3) did not include such boxes.

Figure 3 shows all 30 synthetic images. The relevant ‘figure’ region of each image that represented the food matrix was colored orange so that it resembled the analogous portion of a real image. The smaller, darker objects of different shapes and sizes within the ‘figure’ represent air cells of a real image counterpart. The white rectangular box appearing at the top left of each image in Figure 3 shows the image number (between 1 and 30). Below it and in the same box is the synthetic image’s estimated granularity score (described later). It should be noted that the images that were used as inputs to the DNN did not contain these boxes. Row-1 (top row) of Figure 3 contains LC images, 19, 1, 20, 8, 21, 7, 3, 2. Row-2 contains BO images, 15, 14, 12, 22, 13, 9, 11, 10. Row-3 shows EL images, 27, 30, 29, 26, 28, 23, 24, 25. Row-4 (bottom row) has SC images, 18, 17, 5, 6, 4, 16. The images in each row are arranged in decreasing order of their granularity scores, from best (left) to worst (right).

Each synthetic image was subject to reflection and rotations at intervals of 22.5°, thereby providing L=32 orientations per synthetic image. This was done to obtain a statistically large number of samples from each synthetic image. Accordingly, a total of K×L=960 synthetic images were available for further investigation.

### 2.6. Deep Neural Network

This section describes the main aspects of the enhanced ResNet used in this research. The DNN’s input is a color image denoted as $I\in R^{M\times N\times 3}$ where M×N is the image size. Although pixels of raw images are integers between 0 and 255, they are subject to rescaling and shifts internally in the DNN—an issue that is not addressed here. The output of the last layer is a scalar $\hat{s}$ ($\hat{s}\in R$) representing the estimated fibrosity score of the input image, the corresponding true value being represented as s.

The following passages provide brief descriptions of the layer types and functions.

#### 2.6.1. Convolution Layer

Convolution is very commonly used in digital signal processing as well as in classical image processing. In image processing, it is applied for various spatial operations, such as edge detection, contrast enhancement, and noise removal [57]. A two-dimensional convolution on an array input $X\in R^{SM\times SN}$, using a filter $K\in R^{K\times K}$, yields an output array $Y\in R^{M\times N}$, where K×K is the filter size (K is an odd number) and S is the stride.

For simplicity, let us assume that the horizontal and vertical sizes of array X (SM and SN) are multiples of S, and ignore boundary level image readjustments. Convolution is carried out according to the following expression:

(3) $$\begin{array}{c} Y\left(m,n\right)=\sum_{i,j=-\frac{K-1}{2}}^{\frac{K-1}{2}}K\left(\frac{K-1}{2}-i+1,\frac{K-1}{2}-j+1\right)\cdot X\left(Sm+i,Sn+j\right).\; \end{array}$$

Array indices m, n lie between 1 and M,N in the above expression, which ignores boundary adjustments. It can be seen that convolution reduces the input’s horizontal and vertical sizes by the same factor S. The symbol ‘⋆’ is used to denote the convolution operator, so that the above relationship can be expressed concisely as, Y=K⋆X.

Processing in a convolution layer (Conv) takes place concurrently across multiple input and output channels. Channels have their own, equally sized K×K filters, and identical strides S. Let $c^{\prime }\in \{1,\ldots ,C^{\prime }\}$ be the index of an input channel, and c∈{1,…,C}, that of an output channel [58,59]. The convolved array $I_{c}^{out}$ of output channel c is the summation of $C^{\prime }$ input arrays. Each such array is obtained by convolving input $I_{c^{\prime }}^{in}$ with filter $K_{c^{\prime },c}$ as below:

(4) $$\begin{array}{c} I_{c}^{conv}=\sum_{c^{\prime }=1}^{C^{\prime }}K_{c^{\prime },c}⋆I_{c^{\prime }}^{in}.\; \end{array}$$

Since the input to the proposed DNN is a color image, each color (red, green, blue) may be regarded as an input channel of the first convolution layer, i.e., $C^{\prime }=3$. Downstream image processing layers have significantly more input and output channels. DNNs with multiple convolution layers are routinely used with various food processing applications [25,26,27,60].

A thresholding operation is applied in order to ensure that the scalar elements of the layer’s array output are non-negative. If m, n are array indices, the thresholded output $I_{c}^{out}$ of output channel c is

(5) $$\begin{array}{c} I_{c}^{out}\left(m,n\right)=\max\left\{0,I_{c}^{conv}\left(m,n\right)\right\}.\; \end{array}$$

Thresholding is implemented by means of a ReLU (Rectified Linear Unit) layer [57]. Accordingly, the sequence of operations to obtain any channel output $I_{c}^{out}$ from its input $I_{c^{\prime }}^{in}$ requires that the convolution layer be followed by a ReLU layer. However, it is sufficient for our purpose to assume that thresholding takes place internally within the convolution layer itself, a simpler convention that is often adopted frequently in most of the published DNN literature.

Convolution layers are followed by a pooling layer. The two most commonly used pooling operations are max-pooling and average-pooling. The proposed DNN contains layers for both these types of pooling.

#### 2.6.2. Max-Pooling Layer

Pooling is necessary to lower downstream processing (and training) requirements to computationally tractable levels [61].

The max-pooling layer (MAXPOOL) has the same number of input channels and output channels, C. Its output array $I_{c}^{out}$ is obtained by taking elementwise maxima of K×K pixels of its input $I_{c}^{in}$, in the manner shown below, where overlaps and/or unused pixels determined by the stride:

(6) $$\begin{array}{c} I_{c}^{out}\left(m,n\right)=\underset{-\frac{K-1}{2}\leq i,j\leq \frac{K-1}{2}}{\max}I_{c}^{in}\left(Sm+i,Sn+j\right).\; \end{array}$$

#### 2.6.3. Average-Pooling Layer

Average-pooling replaces elementwise maximums with averages. An average-pooling layer (AVGPOOL) has C input channels, with each carrying a two-dimensional input. However, the layer’s output is a one-dimensional vector that is delivered to a downstream fully connected layer for further processing. Due to this reason, instead of outlining a generic average pooling layer, we focus specifically on the layer incorporated in the proposed DNN. If the input of channel $c\in \left\{1,\ldots ,C\right\}$ is the array $I_{c}^{in}\in R^{M\times N}$, the c^th^ element of the layer’s output vector $x\in R^{C}$ is given by

(7) $$\begin{array}{c} x\left(c\right)=\frac{1}{MN}\sum_{m=1}^{M}\sum_{n=1}^{N}I_{c}^{in}\left(m,n\right).\; \end{array}$$

The proposed DNN contains only a single average-pooling layer as the last image processing stage. Subsequent layers involve high-level vector processing.

#### 2.6.4. Fully Connected Layer

The input to a fully connected layer (FC) is in the form of a one-dimensional array. Its output can be either another array or a scalar. Let $x\in R^{M}$ and $y\in R^{N}$ be the input and output vectors of a fully connected layer. A scalar output can be perceived as a specific case where N=1. The parameters associated with a fully connected layer are a bias vector $b\in R^{N}$ and a weight matrix $W\in R^{N\times M}$.

An activation vector $a\in R^{N}$ is computed internally as a=Wx+b. The FC layer’s output y is determined by applying a (piecewise) continuous, monotonic, and bounded nonlinear function $f:R^{N}\to R^{N}$, i.e., $y=f\left(a\right)$ [62,63]. In the proposed DNN, the output is obtained by imposing a lower threshold on a by means of elementwise ReLu operations.

More specifically, if b(n) is the n^th^ scalar element of b, and $w\left(m\right)\in R^{N}$, the m^th^ column of W, the activation is obtained in the following manner,

(8) $$\begin{array}{c} a\left(n\right)=w^{T}\left(m\right)x+b\left(n\right).\; \end{array}$$

The scalar activation in the RHS of the above expression is thresholded by means of a ReLu nonlinearity, whence the n^th^ scalar output is given as

(9) $$\begin{array}{c} y\left(n\right)=\max\left\{0,a\left(n\right)\right\}.\; \end{array}$$

#### 2.6.5. Residual Connection

The key feature of ResNet is the presence of residual connections, which operate on two different array inputs. One input is the output of the immediately preceding layer. The other input is the output of any other downstream layer. For instance, if there is a residual connection before layer l+1, the two input arrays are the outputs of layers l and l−p (1≤p<l). In this case, we say that the output from the latter “skips” p layers.

The symbols $I_{c}^{{in}_{1}}$ and $I_{c}^{{in}_{2}}$ represent the two input arrays, where the latter skips one or more layers. The output $I_{c}^{out}$ is of the same size as $I_{c}^{{in}_{1}}$, whereas $I_{c}^{{in}_{2}}$, which skips some layers, may be an array of larger size. If the size of $I_{c}^{{in}_{2}}$ exceeds that of $I_{c}^{{in}_{1}}$ and $I_{c}^{out}$, it is subject to down-sampling. In the existing literature on ResNet DNNs, down-sampling is invariably referred to as 1×1 convolution [32], although it does not involve any associated filter.

Down-sampling is applied, when needed, to reduce the size of the skipped input $I_{c}^{{in}_{2}}$ by a factor S, i.e., the stride. This is accomplished by taking regularly spaced samples of $I_{c}^{{in}_{2}}$ at each channel c, to yield another array $I_{c}^{skip}$ whose size matches that of $I_{c}^{{in}_{1}}$,

(10) $$\begin{array}{c} I_{c}^{skip}\left(m,n\right)=I_{c}^{{in}_{2}}\left(Sm,Sn\right).\; \end{array}$$

The output $I_{c}^{out}$ is obtained by adding $I_{c}^{{in}_{1}}$ and $I_{c}^{skip}$,

(11) $$\begin{array}{c} I_{c}^{out}=I_{c}^{{in}_{1}}+I_{c}^{skip}.\; \end{array}$$

When both inputs to the residual connection are equally sized, no down-sampling is needed. This can be viewed as down-sampling with S=1 so that $I_{c}^{skip}=I_{c}^{{in}_{2}}$ when

(12) $$\begin{array}{c} I_{c}^{out}=I_{c}^{{in}_{1}}+I_{c}^{{in}_{2}}.\; \end{array}$$

Residual connections significantly reduce the total number of layers needed by the DNN, which in turn lowers the latter’s training time. If I is the skipped input ($I\equiv I_{c}^{{in}_{2}}$), then $I_{c}^{{in}_{1}}$ is computed by subjecting I to several layers of processing so that $I_{c}^{{in}_{1}}\equiv F\left(I\right)$, where the map $F:R^{SN\times SM}\to R^{N\times M}$ entails some form of nonlinear image processing. To see the usefulness of a residual connection, assume that F(⋅) depicts some spatial blurring operation [32,34]. Replacing I with −I, the result $F\left(I\right)-I$ is an edge image. In other words, this residual connection serves as an edge detector. Deeper layers in the DNN that are involved in image processing can readily extract edge-related information, obviating the need for multiple other image processing layers. This is why the number of layers needed by a ResNet is lower than that of a classical DNN for a comparable task. Fortuitously, this reduction also decreases the overall computational time required to train the ResNet.

In spite of regarding down-sampling as 1×1 convolution, the published research on ResNet architectures typically does not treat residual connections as separate layers—a convention that is adopted throughout this article.

#### 2.6.6. DNN Layout

Ignoring boundary processing, a convolution layer (Conv) can be fully characterized in terms of the number of output channels C, the filter size K×K, and the stride S. This is also the case with a max-pooling layer (MaxPool), where K×K is now interpreted as a window size. So long as the size of the input image is known, a Conv or MaxPool layer’s output size M×N can be readily obtained from S. An average-pooling layer (AVGPOOL) is completely specified in terms of the size of its output vector C. In a similar manner, the output size N alone suffices to describe the layout of any fully connected layer (FC). The only determinant of down-sampling is the stride S.

**Figure 4:**
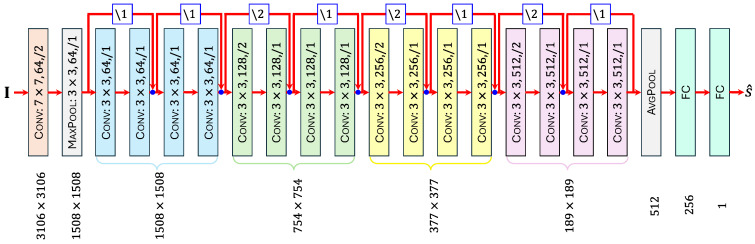
**Layout of Proposed DNN.** The DNN’s input image $I\in R^{6032\times 6032\times 3}$ may be either a preprocessed real image or a synthetic image in any spatial orientation. The output $\hat{s}\in [1,10]$ is the corresponding estimated fibrosity score. Colored rectangles are the network’s layers. Layers within each block have the same color.

Figure 4 illustrates the architecture of the modified ResNet that was developed for this research. Layers are represented as colored rectangles. Parametric constants of each Conv and MAXPOOL layer are provided inside the rectangles, and in the format K×K,C,\S, which is consistent with published research. The layer’s output size is shown below it.

The DNN’s input image I undergoes several layers of image processing. After the initial Conv layer and MAXPOOL layer, downstream image processing layers are grouped into four blocks, and each such block comprises four Conv layers with identical output sizes. The layers in each block are shown as rectangles with the same color. All connections are shown as red arrows. The strides of residual connections (Equation (8)) are provided in the format \S and enclosed within small blue squares. The pixelwise additions involved in Equations (9) and (10) are depicted as blue dots.

Two FC layers follow the final image processing AVGPOOL layer. They are the only trainable layers in the DNN. The second FC layer, which contains a single neuronal unit, determines the overall DNN output, which is the estimated quality score $\hat{s}$.

#### 2.6.7. DNN Training

Only the two FC layers of the DNN were trained. Samples were drawn randomly from some dataset S and divided in the standard ratio of 85:15 into two: a training set $S_{t}$ and a test set $S_{e}$, where $S=S_{t}\cup S_{e}$, $S_{t}\cap S_{e}=\emptyset$, $\left|S_{t}\right|=0.85\left|S\right|$, and $\left|S_{e}\right|=0.15\left|S\right|$.

Referring to Equation (2), the last field in $S_{t}$, which was of the form ${\left\{I_{i,l},s_{i,l}\right\}}_{l=1\ldots L}$, was used to train the DNN. An image $I_{i,l}$ was drawn at random to serve as the input to the DNN, and its output ${\tilde{s}}_{i,l}$ was the corresponding estimated score. The purpose of training was to adjust the FC weights and biases until the estimates were as close as possible to the real subjects’ scores. The sum squared error loss shown below was used for minimization:

(13) $$\begin{array}{c} E=\sum_{i,l\in S_{t}}{\left(s_{i,l}-{\tilde{s}}_{i,l}\right)}^{2}.\; \end{array}$$

Sum squared error loss functions are routinely used in training algorithms for regression [19]. Current DNN training algorithms add regularization terms to the loss [64].

Details of the training algorithm are not provided here, as they are standardized aspects that are built-in within Pytorch [44] and the Torchvision package [65]. It suffices for our purpose to merely mention that a form of stochastic gradient descent was applied to minimize the loss in Equation (13). An epoch is a single pass through all training samples. The weights of the FC layers were updated incrementally through several epochs, with an up-to-date learning method based on the stochastic gradient descent rule [66],

(14a) $$\begin{array}{c} W=\left(1-\eta \right)W+\eta \nabla_{W}E, \end{array}$$

(14b) $$\begin{array}{c} b=\left(1-\eta \right)b+\eta \nabla_{b}E. \end{array}$$

Although the learning rate η in the above is depicted as a constant, in reality it varies across layers and is progressively reduced with training epoch.

State-of-the-art DNN learning algorithms offer several improvements over classical stochastic gradient descent, such as batch normalization, dropout, and other schemes. For further details, the reader is referred elsewhere [14]. These features are an integral part of Pytorch software. The code internally sets aside a proportion of samples from $S_{t}$ for validation. Suitable features were selected during the DNN training.

The DNN was trained using the ADAM optimizer [44,65]. During training, dropout layers were added to the FC layers. As dropout layers were not required beyond the training stage, they are not shown in Figure 4. Regularization techniques were employed to improve generalization and prevent overfitting.

The significant training parameters were as follows. The learning rate was kept at $\eta =10^{-4}$. The weight decay (L_2_ regularization) was set to 0.001. The dropout rate after the FC layers was set to 0.5. Additionally, a learning rate scheduler (ReduceLROnPlateau) was applied to reduce the learning rate by a factor of 0.5 whenever the validation loss would not decrease for three consecutive epochs. Early stopping was implemented with a patience of 20 epochs, ensuring that training halted once the performance began to plateau. The DNN was trained for up to 5000 epochs, with batch sizes of 8 and 32 for the training and validation datasets. Other secondary aspects of DNN training, which did not play any significant role, therefore have not been addressed in this article.

### 2.7. Statistical Metrics

In accordance with prior research [19], the statistical metrics that were adopted in this research fall under three categories: (*i*) error norm metrics ($E_{2}$, $E_{1}$), (*ii*) goodness-of-fit metrics ($R^{2}$, C), and (*iii*) linear regression metrics (r, m, b). The goodness-of-fit metrics use score means whose underlying expressions are as given below:

(15a) $$\begin{array}{c} \bar{s}={\left|S_{e}\right|}^{-1}\sum_{i\in S_{e}}{\bar{s}}_{i},\; \end{array}$$

(15b) $$\begin{array}{c} \bar{\tilde{s}}={\left|S_{e}\right|}^{-1}\sum_{i\in S_{e}}{\bar{\tilde{s}}}_{i}.\; \end{array}$$

Depending on the dataset, the quantity ${\bar{s}}_{i}$ may refer either to one of the two subject’s scores, ${\bar{s}}_{i}^{A}$ or ${\bar{s}}_{i}^{B}$, or to their weighted mean. Brief descriptions of each category follow.

(*i*) Error Norm: The two norm-based errors used in this research are the mean squared error $E_{2}$, and the averaged absolute error $E_{1}$. They are normalizations of the squared L_2_ norm (Euclidean distance) and they L_1_ norm (Manhattan distance). The errors are defined as below:

(16a) $$\begin{array}{c} E_{2}={\left|S_{e}\right|}^{-1}\sum_{i\in S_{e}}{\left({\bar{s}}_{i}-{\bar{\tilde{s}}}_{i}\right)}^{2},\; \end{array}$$

(16b) $$\begin{array}{c} E_{1}={\left|S_{e}\right|}^{-1}\sum_{i\in S_{e}}\left|{\bar{s}}_{i}-{\bar{\tilde{s}}}_{i}\right|.\; \end{array}$$

The ideal case, i.e., when the estimates are accurate (${\bar{s}}_{i}={\bar{\tilde{s}}}_{i}$), $E_{2}=0$ and $E_{1}=0$.

(*ii*) Goodness-of-Fit: The coefficients of determination $R^{2}$, and correlation C, are as shown in the expressions below:

(17a) $$\begin{array}{c} R^{2}=1-{\left(\sum_{i\in S}{\left({\bar{s}}_{i}-\bar{s}\right)}^{2}\right)}^{-1}\sum_{i\in S}{\left({\bar{s}}_{i}-{\bar{\tilde{s}}}_{i}\right)}^{2},\; \end{array}$$

(17b) $$\begin{array}{c} C={\left(\sum_{i\in S}{\left({\bar{s}}_{i}-\bar{s}\right)}^{2}\sum_{i\in S}\left({\bar{\tilde{s}}}_{i}-\bar{\tilde{s}}\right)\right)}^{-\frac{1}{2}}\sum_{i\in S}\left({\bar{s}}_{i}-\bar{s}\right)\left({\bar{\tilde{s}}}_{i}-\bar{\tilde{s}}\right).\; \end{array}$$

The quantities $\bar{s}$ and $\bar{\tilde{s}}$ in the RHS of the above expressions are obtained from Equation (15). The ideal values of the coefficients are $R^{2}=1$ and C=1.

(*iii*) Linear Regression: Linear regression is applied with the y-intercept constrained to zero to obtain a straight line of slope r passing through the origin. It is also applied without this constraint to obtain a line with slope m and y-intercept b. Mathematically,

(18a) $$\begin{array}{c} r=\underset{r}{\mathrm{arginf}}\sum_{i\in S}{\left(r{\bar{s}}_{i}-{\bar{\tilde{s}}}_{i}\right)}^{2},\; \end{array}$$

(18b) $$\begin{array}{c} m,b=\underset{m,b}{\mathrm{arginf}}\sum_{i\in S}{\left(m{\bar{s}}_{i}+b-{\bar{\tilde{s}}}_{i}\right)}^{2}. \end{array}$$

The best outcome is when the slopes are r=1 and m=1, and the y-intercept is b=0.

## 3. Results

### 3.1. Results: Real Images

In order to deal with intrinsic differences between subjects A and B, the extended ResNet was trained separately three times, using the individual datasets $S^{A}$, $S^{B}$ of subjects A and B, as well as with their combined dataset, $S^{A}\cup S^{B}$. Accordingly, the relevant dataset in an experiment is $S\in \left\{S^{A},S^{B},\;S^{A}\cup S^{B}\right\}$. As described earlier (Section 2), S was divided randomly into a training dataset $S_{t}$ and a test dataset $S_{e}$.

**Figure 5:**
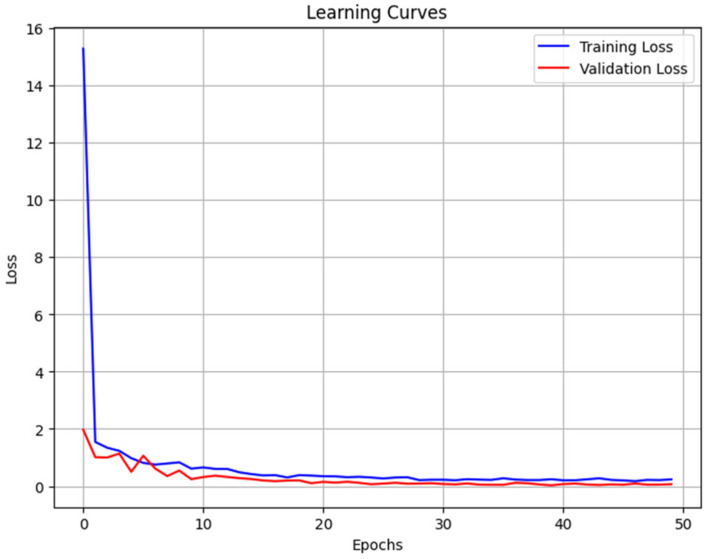
**Learning Curves.** The loss E per epoch when the DNN was trained with the combined training dataset $S_{t}^{A}\cup S_{t}^{B}$.

Figure 5 shows how the loss, as defined in Equation (13), decreased steadily as the ResNet was trained with $S_{t}^{A}\cup S_{t}^{B}$, which is internally split into training and validation subsets. Training and validation losses are shown as blue and red colored curves. In order to avoid redundancy, similar plots with $S_{t}^{A}$ and $S_{t}^{B}$ are not included herein.

**Figure 6:**
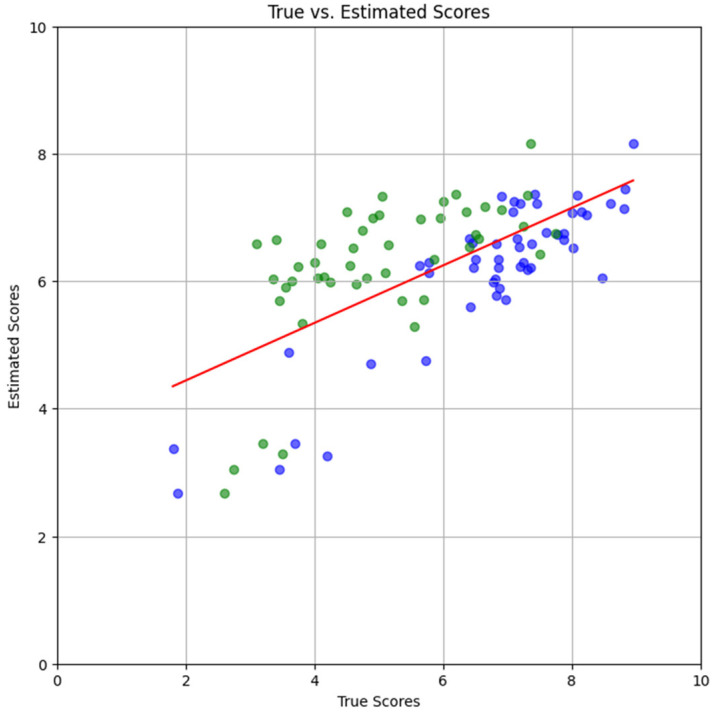
**Combined Scatter Plot**. Points in the scatter plot pertain to subjects *A* (green) and *B* (blue). Also shown is the regression line (red) from the combined dataset. Only the test dataset $S_{e}^{A}\cup S_{e}^{B}$ was used in this figure.

**Figure 7:**
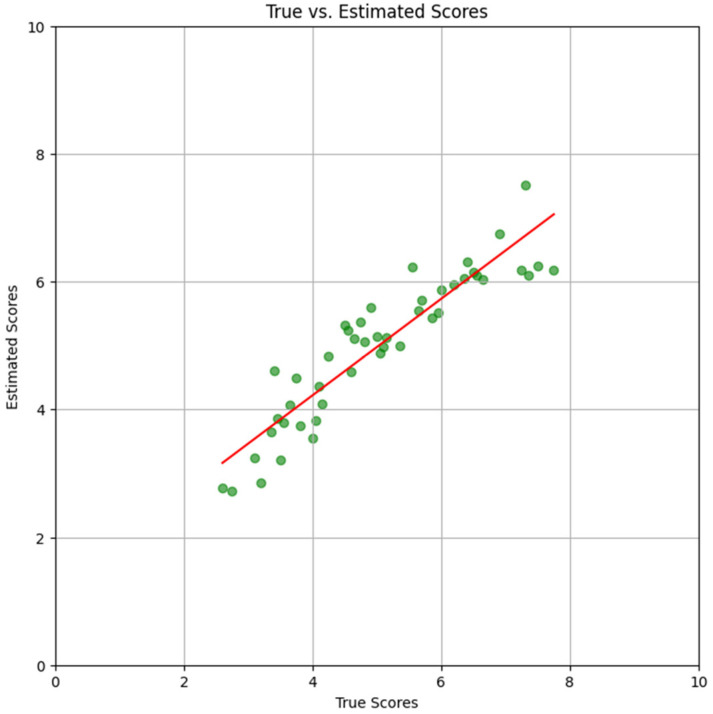
**Scatter Plot (Subject *****A*****)**. Scatter plot and regression line with subject *A* scores, using the same color scheme as that in Figure 6. Only the test dataset $S_{e}^{A}$ was used.

**Figure 8:**
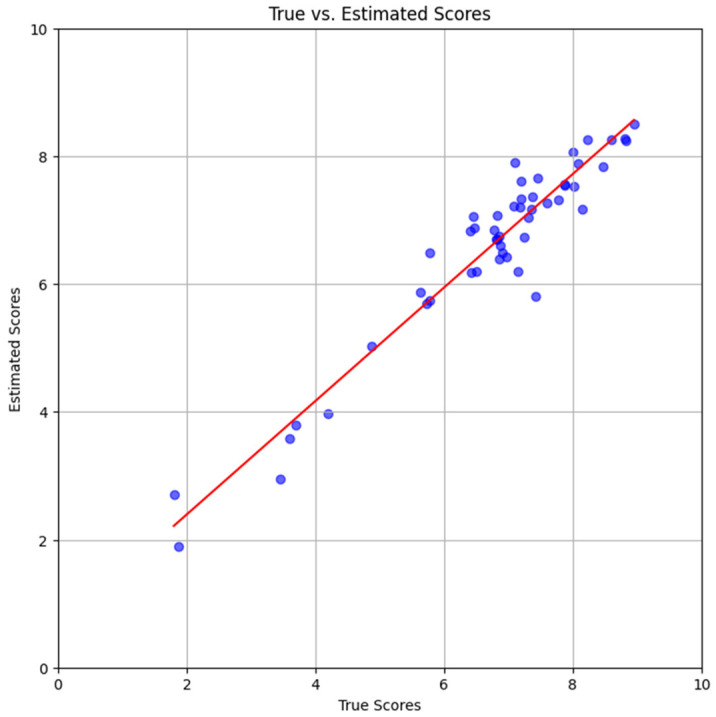
**Scatter Plot (Subject *****B*****)**. Scatter plot and regression line with subject *B* scores, using the same color scheme as that in Figure 6. Only the test dataset $S_{e}^{B}$ was used.

The scatter plots in Figure 6, Figure 7 and Figure 8 show results with only the test dataset $S_{e}$. Referring to Equation (2), for each image $I_{i}$ in $S_{e}$, all L=8 orientations ${\left\{I_{i,l}\right\}}_{l=1\ldots L}$ were inputted separately to the trained DNN; their corresponding DNN outputs were the estimated scores ${\left\{{\tilde{s}}_{i,l}\right\}}_{l=1\ldots L}$. The mean estimate was obtained as

(19) $$\begin{array}{c} {\bar{\tilde{s}}}_{i}=\frac{1}{L}\sum_{l=1}^{L}{\tilde{s}}_{i,l}.\; \end{array}$$

Figure 6 shows the outcome when the DNN was trained with the combined dataset $S=S^{A}\cup S^{B}$. The points in this scatter plot correspond to true scores (*x*-axis) vs. estimated scores (*y*-axis). They are shown in blue and green colors for subjects A and B, so that the 2-D coordinate of a blue point is $\left({\bar{s}}_{i}^{A},{\bar{\tilde{s}}}_{i}\right),$ whereas that of a green point is $\left({\bar{s}}_{i}^{B},{\bar{\tilde{s}}}_{i}\right)$. The regression line ${\bar{\tilde{s}}}_{i}=m{\bar{s}}_{i}+b$ is shown in red, where ${\bar{s}}_{i}$ are the mean scores, weighted in proportion to sample size:

(20) $$\begin{array}{c} {\bar{s}}_{i}={\left(P^{A}+P^{B}\right)}^{-1}\left(P^{A}{\bar{s}}_{i}^{A}+P^{B}{\bar{s}}_{i}^{B}\right).\; \end{array}$$

It can be observed from Figure 6 that there were discernible differences in the true scores of subjects A and B. Subject A’s scores were relatively uniformly distributed, whereas subject B’s scores followed a distribution that was skewed in the positive (rightward) direction. In addition, the latter’s scores were scattered over a wider range than those of subject A. As a result of these dissimilarities, the linear regression line (red color) had a slope of only m=0.45 and a relatively high y-intercept of b=3.54.

Figure 7 shows the scatter plot when the DNN was trained using dataset $S_{t}^{A}$. The linear regression line (red) is also shown. Its slope (m=0.76) and y-intercept (b=1.20) showed a marked improvement in the DNN’s performance in comparison to Figure 6.

Figure 8 depicts the outcome when the DNN was trained with dataset $S_{t}^{B}$. The slope (m=0.89) and y-intercept (b=0.62) of the regression line were significantly better than those in Figure 6. Figure 6 and Figure 7 clearly indicate that the DNN’s estimates were more accurate when it was trained separately with only a single subject’s dataset.

Table 1 shows all statistical performance metrics, with those in each category placed in adjacent columns. As before, it can be observed that training the DNN with individual datasets yielded better estimates in comparison to those from their combined dataset.

### 3.2. Results: Synthetic Images

Since synthetic images were used to explain the DNN’s outputs, all real images in $S^{A}$ and $S^{B}$ were available for training. As discussed earlier (Figure 6 and Figure 7), human subject scores were unevenly distributed. In the combined training dataset $S^{A}\cup S^{B}$, just two scores were lower than 2, albeit marginally, only a few above 8, and none exceeded 9. Early experiments yielded estimates that were heavily concentrated around the middle. In order to broaden the range of DNN estimates, the combined dataset had to be subject to further augmentation. Only nine equally spaced intervals were considered, 1–2, 2–3, 3–4, 4–5, 5–6, 6–7, 7–8, 8–9. Samples from intervals with lower frequencies were picked at random and duplicated. This process was repeated until all intervals had the same number of samples. Weighted means scores as in Equation (20) were used as target output scores. All other aspects of DNN training were as discussed earlier.

All L=32 orientations $I_{i,l}$ of each synthetic image $I_{i}$ were used as inputs to the trained DNN, whose outputs are the corresponding estimated scores ${\tilde{s}}_{i,l}$. Table 2 provides summary statistics of the L=32 scores (columns 3–8).

The two left-most columns (columns 1, 2) show image numbers and categories—LC (“large circle”), BO (“box”), EL (“ellipse”), and SC (“small circle”). The rows in Table 2 pertain to synthetic images $I_{i}$ and are arranged in decreasing order of the means of their estimated fibrosity scores ${\bar{\tilde{s}}}_{i}$ across all 32 orientations that were obtained using Equation (19). The table’s headers are self-explanatory.

Table 2 is best interpreted when viewed alongside Figure 3. The DNN’s estimated fibrosity score of each synthetic image was interpreted in terms of the shape, size and orientation of the food matrix (orange color) of its enclosed smaller air cells (dark brown), as well as how many of the latter were inside the matrix. It should be emphasized that the interpretations discussed in this section are of a simple and informal nature, and that they were deduced only through careful, visual inspection. Moreover, the terms ‘interpretation’ and ‘explanation’ have been used interchangeably.

Table 2 (columns 5, 6) shows that the range of scores across all 30 rotations of an image was acceptably lower than its average (column 3), illustrating that the input image’s spatial orientation did not adversely affect the DNN’s output. Image no. 1 represents the worst-case scenario. The difference between its maximum and minimum scores (6.1682, 3.8545) is 48.28% of its average score (4.7921), which is high. In image no. 4, the standard deviation (0.4451) is 13.79% of the average (3.2264)—this is highest ratio of all 30 images. It can be seen from Figure 3 that both images are in the category of small circles (‘SC’). A possible explanation is that due to their smaller sizes, the DNN was unable to acquire enough cues for more precise estimates.

The average scores shown in Table 2 (column 3), as well as in Figure 3 (small, white rectangles), follow a remarkably consistent pattern. For instance, in Row-4 (‘SC’) in Figure 3, the leftmost image (no. 18) with the highest score of 5.9922 had eight elongated air cells inside the food matrix. The next image (no. 17) had only five such ones that were somewhat wider; consequently, it received a lower score of 4.3066. The same logical trend could be seen throughout this row, as well as in the figure’s Row-1 (‘LC’) and Row-2 (‘BO’).

Row-3 (‘EL’) of Figure 3 was more interesting. The first four images (nos. 27, 30, 29, 26) received very similar scores between 6.1946 and 6.0889. This is because each image incorporated eight similarly shaped elongated air cells. Although the fifth image in this row (no. 28) also had eight such cell structures, they were relatively not as long. The rightmost image in Row-3 (no. 25), which had only circular air cells, was assigned the lowest score of 3.4792. The first two images in Row-1 (nos. 19 and 1) did not seem to follow this trend; despite having only seven long air cells, the leftmost image received a higher score of 4.9973 than the other image which had eight of them; the latter’s estimated score average was 4.7921. Although a complete verbal explanation is impossible, it was surmised that image 19 scored better as its air cells were relatively longer than those in image no. 1.

Comparisons across the rows in Figure 3 shed further insights into how the DNN was able to provide fibrosity estimates. The scores of leftmost images in the rows were 4.9974 (no. 19), 6.7716 (no. 15), 6.1976 (no. 27), and 5.9922 (no. 18). Although image no. 19 had the longest cells, due to the large size (area) of the food matrix, the cells provided a sparser coverage. It could be seen that the rightmost images in the rows only had circular air cells. Accordingly, they were assigned the row-wise lowest scores of 1.7277 (no. 2), 3.7630 (no. 10), 3.4792 (no. 25), and 2.6468 (no. 16).

Image no. 2 was assigned the lowest score in the entire set of 30 images. The reason for this was evident—despite its large size, the seven cell structures contained in it provided proportionately very little coverage. The difference between the scores of image nos. 10 and 25 was small. Visually they also looked alike. Image no. 16 scored lower than either, which appeared to digress from the overall pattern. A plausible explanation that we put forth is that this image had a comparatively lower number of circular air cells within the food matrix.

## 4. Discussion

The salient contributions of this research are threefold and are as outlined in the following paragraphs.

Firstly, it was demonstrated that a DNN can be successfully applied to estimate granularities from input images in the manner of human experts. This was evidenced from the results with real images in Section 3. In spite of limited image samples and prior human scores, the DNN could be trained for this purpose, whose accuracy is reflected through multiple statistical performance metrics. This task was accomplished using a suitable ResNet-18 layout with an additional layer, combined with appropriate spatial image preprocessing, data enhancement, and transfer learning. Although ResNets are routinely used for similar applications, to the best of the authors’ knowledge, the DNN in this research is the first to be developed for the regression task of estimating fibrosities of meat analogs.

Next, close examination of the differences between the subjects’ scoring pattern and the DNN’s significantly better performance when separately trained with each subject scores suggested that the DNN could integrate into its estimation parameters more subtle aspects of human scores. Although the outcome is not conclusive, the authors believe that it would be worthwhile to extend this study with the collection and statistical analysis of more subject scores and how they correlate with various image properties, as well as to account for the extent of implicit, perceptual bias.

Lastly, the outcome of the experiment with synthetic images is noteworthy. In the authors’ views, the DNN’s estimated granularity scores followed a remarkably consistent pattern that was amenable to simple, straightforward interpretation in terms of features of the input images. The study strongly suggests that the DNN’s estimation scheme was based on the extent of coverage provided to the food matrix by the air cells contained in it, the number of them present and their elongations.

Needless to say, this research is not without limitations. Although it highlights the feasibility of using such DNNs to assess the granularities of extruded plant-based meat products from camera images, sans human intervention, all real images used here were obtained solely by the present team. An in-depth analysis of human assessment would have been possible by collecting subject scores from a larger group of human experts. The DNN’s estimates was interpreted through visual observations. Quantifying the matrix and cell properties in the synthetic images would have allowed for more mathematically rigorous interpretation analysis.

## 5. Conclusions

This research demonstrates the effectiveness of the proposed DNN, which is an extension of ResNet-18, in estimating the fibrosities of plant-based meat analogs from camera images. It was shown that with only a reasonably limited amount of data and appropriate augmentation, the DNN could be trained to provide estimates with a high degree of accuracy. Simulation results with real images illustrate that this DNN was capable of incorporating perceptual elements present in human assessment of plant-based meat quality.

Human scores were used only for the DNN’s training and evaluation; considering the possibility that some deeper aspects of human assessment may be dauntingly complex for this research [67], their underlying perceptual basis remains outside the scope of this study. This is unlike the approach taken in [10], where computer vision algorithms were applied to obtain a set of prespecified textural attributes, which were correlated with human visual inspection. Instead of selecting *a priori* only some features for investigation, a holistic approach has been adopted here. Only limited fine-tuning with additional data is needed to customize a DNN for other plant-based meat analogs, as well as for other desired textural features. Traditional computer vision approaches do not offer this kind of flexibility.

Analysis of the DNN’s scores with synthetic image inputs illustrates that an undue amount of experimental data is not needed to elicit high-performance accuracy. This task can be achieved by selecting a suitable layout (e.g., the extended ResNet layout proposed here) and appropriate data preprocessing, augmentation, and transfer learning steps. Furthermore, interpreting this experiment’s outcome, suggests that this scheme endowed the DNN with the ability to discern intrinsic, perceptual differences in human experts and be free of bias [52].

Future research can be pursued along several directions. Fibrosities of plant-based meat products are influenced by multiple spatial elements present in their food matrices and air cells. All these features can be quantified using suitable image segmentation and labeling algorithms [68]. While they can be integrated into a single, empirical fibrosity measure per image sample, the authors’ plan to explore using Pareto optimality—a concept widely used in multicriteria decision-making research [69]—as an alternative criterion to assess fibrosities. Active learning can be investigated for continual re-adaptation under changing external conditions [70].

Computational tools are available for the purpose of DNN explainability [54,55]. Many of these methods are model-agnostic, i.e., they treat machine learning models as black boxes. However, other methods, which are specific to DNNs, are also available [31,71]. They are used to impart explainable elements while training the DNN. A suitable method can be adopted to train a ResNet with the proposed layout, such that its input–output mapping would be more tractable for explainability analysis.

Lastly, research should also be aimed towards the fully automated optimization of extrusion process control parameters. This goal would require the use of reinforcement learning. Up-to-date deep reinforcement learning models, which are equipped with one or more DNNs, have met with a great deal of success in a wide variety of complex, real-world applications [15,20,23,72].

## Figures and Tables

**Table 1:** Statistical metrics with real image datasets.

Test Set $S_{e}$	Error Norm		Goodness of Fit		Regression		
	$E_{2}$	$E_{1}$	$R^{2}$	C	r	m	b
$S_{e}^{A}\cup S_{e}^{B}$	1.7198	1.0471	0.4155	0.6676	1.0038	0.45	3.54
$S_{e}^{A}$	0.3062	0.4166	0.8387	0.9218	0.9772	0.76	1.20
$S_{e}^{B}$	0.2229	0.3581	0.9122	0.9594	0.9751	0.89	0.62

**Table 2:** Scores with synthetic images.

No.	Cat.	Estimated Score (32 Orientations)				
		Avg.	Med.	Min.	Max.	Std.
15	BO	6.7716	6.7152	5.8834	7.5957	0.5084
14	BO	6.5857	6.4917	6.1085	7.2480	0.3206
12	BO	6.5242	6.3535	5.7271	7.7302	0.5639
27	EL	6.1946	6.1781	5.5981	6.6784	0.3570
30	EL	6.1868	6.2695	5.4897	6.8186	0.3954
29	EL	6.1142	6.1447	4.9612	6.8343	0.4535
26	EL	6.0889	6.0014	5.5629	6.7248	0.3779
28	EL	6.0808	6.0857	5.5080	6.8620	0.3434
22	BO	6.0405	5.9445	5.3056	6.9674	0.4756
18	SC	5.9922	5.9873	5.5479	6.4865	0.2265
23	EL	5.9096	5.9609	5.0529	6.6452	0.4213
13	BO	5.3727	5.3345	4.7956	6.1312	0.3838
9	BO	5.2419	5.0952	4.2173	6.5956	0.8068
19	LC	4.9973	4.9878	4.1453	5.8929	0.5149
1	LC	4.7921	4.7188	3.8545	6.1682	0.6730
24	EL	4.4814	4.4687	4.1215	4.8845	0.2288
11	BO	4.4190	4.1944	3.8615	5.5724	0.5932
17	SC	4.3066	4.2440	3.8008	4.8826	0.3150
20	LC	4.1275	4.2039	3.3024	5.2319	0.5109
8	LC	3.9899	3.7618	2.6536	5.4541	0.7026
21	LC	3.9099	3.9401	3.2163	4.3710	0.3511
10	BO	3.7630	3.6224	3.3140	4.6773	0.4312
5	SC	3.6119	3.4995	3.1894	4.4612	0.3747
6	SC	3.5703	3.6371	2.8441	4.0011	0.3119
25	EL	3.4792	3.5238	3.1798	3.7570	0.2044
4	SC	3.2264	3.1495	2.5932	4.1181	0.4451
16	SC	2.6468	2.6389	2.3321	2.9631	0.1437
7	LC	2.5946	2.5982	2.3745	2.8544	0.1170
3	LC	2.4213	2.4301	1.6123	3.2826	0.4522
2	LC	1.7277	1.7256	1.5278	1.8731	0.0872

## Data Availability

The original contributions presented in the study are included in the article, further inquiries can be directed to the corresponding author.
